# Evolocumab for early reduction of LDL-C levels in patients with acute ischemic stroke: a randomized controlled trial

**DOI:** 10.3389/fneur.2024.1454608

**Published:** 2024-12-23

**Authors:** Xiaohui Qiu, Yeyuan Liang, Yunfei Wei, Mengru Lu, Yujia Mei, Shiting Tang, Jian Tang, Jinyu Liang, Junli Liang

**Affiliations:** Department of Neurology, The Second Affiliated Hospital of Guangxi Medical University, Nanning, China

**Keywords:** PCSK9 inhibitor, evolocumab, ischemic stroke, statins, LDL-C

## Abstract

**Background:**

Low-density lipoprotein cholesterol (LDL-C) has been determined as an established risk factor for acute ischemic stroke (AIS). Despite the recommendation for in-hospital initiation of high-intensity statin therapy in AIS patients, achieving the desired target LDL-C levels remains challenging. Evolocumab, a highly effective and quickly acting agent for reducing LDL-C levels, has yet to undergo extensively exploration in the acute phase of AIS. The aim was to assess the LDL-C reduction efficacy and safety of early application of evolocumab during the in-hospital phase of AIS patients.

**Methods:**

An unblinded, single-center, prospective randomized controlled trial involving hospitalized AIS patients was conducted in the Second Affiliated Hospital of Guangxi Medical University in China. Patients were randomly assigned 1:1 to receive evolocumab 420 mg every 4 weeks or not, on top of standard of care (SOC) treatment (atorvastatin 40 mg/day and ezetimibe 10 mg/day), administered in-hospital until after 8 weeks. The primary outcome was the absolute change of LDL-C levels and the rate of achieving targeted lipid control at 8 weeks.

**Results:**

Totally, 120 patients were recruited from January 2023 to December 2023. Mean LDL-C levels decreased from 3.15 mmol/L to 0.85 mmol/L in the evolocumab group, and from 3.17 mmol/L to 2.22 mmol/L in the control group, with the difference in mean change from baseline was −1.37 [95% confidence interval (CI) = −1.70 to −1.04, *p* < 0.001] at week 8. The rate of patients achieving targeted LDL-C <1.4 mmol/L was 81.67% in evolocumab group as compared with 13.33% in control group. Adverse events were similar in both groups.

**Conclusion:**

Our study indicated that evolocumab added to high-intensity statin and ezetimibe therapy resulted in substantial reduction in LDL-C levels in early AIS patients and was well tolerated.

**Clinical trial registration:**

ClinicalTrials.gov, identifier NCT05697185.

## Introduction

Stroke remains a leading cause of morbidity and mortality worldwide, with ischemic stroke comprising a significant portion of these cases (1). In recent years, as the pathophysiological mechanism of stroke has been studied in greater depth, it has been discovered that dyslipidemia, especially elevated low-density lipoprotein cholesterol (LDL-C), is an important risk factor for the occurrence and recurrence of intracranial atherosclerotic ischemic stroke (2). A study encompassing more than 11,000 patients diagnosed with coronary heart disease (CHD) has revealed a significant 14% increase in the relative risk of verified ischemic stroke or transient ischemic attack for every 40 mg/dL (1.03 mmol/L) increase in LDL-C levels (3). Numerous studies have conclusively shown that lipid-lowering therapies are highly efficacious in both primary and secondary prevention of acute ischemic stroke (AIS) (4). In a comprehensive meta-analysis of 21 global randomized trials for LDL-C-lowering drug treatment, a 1 mmol/L reduction in LDL-C can decrease the risk of ischemic stroke by approximately one-fifth (5). It was further demonstrated that reducing LDL-C to <1.4 mmol/L or 50% of baseline level was more effective in preventing ischemic stroke recurrence and improving clinical outcomes (6, 7).

Statin treatment was the cornerstone of dyslipidemia management and had an established role in AIS prevention (8). The recently published 2021 AHA/American Stroke Association guideline for the prevention of recurrent stroke advised that, for patients suffering from noncardioembolic ischemic stroke and exhibiting an LDL-C level exceeding 100 mg/dL, the administration of atorvastatin at a dosage of 80 mg daily was indicated as a measure to mitigate the risk of recurrent stroke (9). While according to the EUROASPIRE III survey, roughly two-thirds of patients failed to attain the prescribed target levels of LDL-C in view of the delayed onset of action of statins (10). The current paradigm for lipid management favoured a stepwise approach consisting of early initiation of high-intensity statin, followed by subsequent addition of ezetimibe, and ultimately consideration of proprotein convertase subtilisin/kexin type 9 (PCSK9) inhibitor treatment if LDL-C levels remain elevated (11, 12). In the lower-target arm of the treating stroke to target trial, an LDL-C level of 65 mg/dL was attained in just 24% of patients within group who received high-intensity statins, whereas a significantly higher proportion of patients in group who were administered a combination of statins plus ezetimibe with 41% (13). However, high-intensity statins therapies were associated with a heightened risk of hemorrhagic stroke attributed to its off-target pharmacological effects. And it was observed that there was no diminished risk of recurrent stroke among patients without evidence of atherosclerosis (14, 15). Fixed-dose combinations of statins and ezetimibe were also the standard of care (SOC) treatment for patients with atherosclerotic cardiovascular disease and elevated LDL-C levels (16).

Evolocumab was a PCSK9 antibody and a new class of drug that rapidly and effectively lower LDL-C levels (17). In the FOURIER trial, a study on 27,564 patients with stable atherosclerosis and elevated risk, evolocumab achieved a 59% reduction in LDL-C (absolute reduction of 1.45 mmol/L) when administered alongside moderate- or high-intensity statins. This significant reduction in cardiovascular events was observed over a median follow-up period of 2.2 years (18). They exhibited significantly less association with identified off-target antithrombotic effects (19). Furthermore, an in-depth analysis of patients receiving evolocumab indicated a substantial reduction in both first and total strokes (including recurrent) of any type and a significant decrease in the degree of dependency poststroke (20). However, this prespecified subgroup analysis was derived from the FOURIER trial, which was powered based on all eligible patients for a composite cardiovascular end point. Consequently, the power to explore individual secondary end points and subgroup effects among patients with prior ischemic stroke was moderate. Evolocumab also demonstrated anti-inflammatory properties, contributing to the stabilization of atherosclerotic plaques and reducing infarction volume, as evidenced by in-vitro and animal studies (21, 22). These pleiotropic effects have also been observed in small human studies, with intravenous ultrasound revealing regression of atherosclerotic plaques (23, 24).

The findings suggested that ischemic stroke patients may benefit from reduction of LDL-C levels below current therapeutic targets by adding evolocumab additionally. However, there is currently few study results worldwide on the use of evolocumab in AIS patients. The inquiry as to whether evolocumab has the potential to reduce LDL-C levels in AIS patients during the early stages of disease is deserved of thorough investigation. Besides, it also has attracted much attention for its safety in early application in AIS patients.

This study attempts to validate the cholesterol, especially for LDL-C, lowering efficacy and safety of evolocumab initiated during the in-hospital phase of AIS patients. We hypothesized that compared to the SOC treatment, adding use of evolocumab among AIS will have a better reduction of LDL-C with well tolerated.

## Methods

### Study design

This study was an unblinded single-center, prospective, two-arm, randomized control study with 1:1 allocation ratio. The research protocol was performed following the Declaration of Helsinki and approved by the Ethical Committee of the Second Affiliated Hospital of Guangxi Medical University (KY-0012). Written informed consent was obtained from each participant, and they were free to withdraw from the study at any point. This study was registered in a publicly accessible clinical trials registry (ClinicalTrials.gov, ID: NCT05697185).

### Participants

A total 120 eligible consecutive AIS patients hospitalized at the department of neurology, the Second Affiliated Hospital of Guangxi Medical University from January 2023 to December 2023 were planed recruiting in this cohort. Inclusion criteria were as follows: (1) aged 18 to 80 years; (2) hospitalized with symptom onset <24 h; (3) atherosclerotic origin; (4) LDL-C levels upon hospital admission had to be higher than guideline-recommended targets of 1.4 mmol/L (55 mg/dL) whether prior statin therapy or not; (5) pre-stroke modified Rankin Scale ≤2; (6) ability to understand the requirements of the study and to provide informed consent. Exclusion criteria were as follows: (1) coexistence of intracranial hemorrhagic disease; (2) cardioembolic stroke (valvular heart disease, atrial fibrillation or aortic arch atheroma); (3) patients treated with PCSK-9 inhibitors before; (4) severe hepatic or renal dysfunction; (5) patients with modified Rankin Scale >4. AIS was defined by World Health Organization criteria as a sudden focal neurologic deficit persisting longer than 24 h and confirmed by brain CT or MRI. Demographic data and baseline clinical characteristics were collected by trained study staff through in-person interviews of patients and a review of electronic medical records at the time of enrolment.

### Interventions

The study drug was administered as promptly as feasible at baseline, ensuring administration within a maximum of 24 h following randomization. In patients scheduled to undergo digital subtraction angiography, either standalone or accompanied by vascular intervention therapy, it was preferred to administer evolocumab prior to digital subtraction angiography whenever possible. However, administration after digital subtraction angiography was also deemed acceptable. Blood samples were collected in the early morning of the day following admission to measure fasting lipids using immunoturbidimetric assay at baseline. All patients received SOC treatment throughout the study, which defined as the standard lipid-lowering treatment including atorvastatin 40 mg/day and ezetimibe 10 mg/day. All patients were randomly assigned 1:1 ratio by random number table method to additionally receive evolocumab 420 mg every 4 weeks or not by the researchers in the study team. The participants were not blinded to the intervention allocation due to the nature of intervention. Adjustments to statin therapy or addition of other lipid-lowering therapies were discouraged throughout the study. Enrolled patients were treated for the AIS event in accordance with current guidelines, including medical treatment with or without stent implantation or balloon inflation. The second study drug administration was performed during a visit at 4 weeks, and the final clinical visit was scheduled at 8 weeks. Patients undergo follow-up through either telephonic or face-to-face consultations at the outpatient facility. All clinical and medication records were extracted by dedicated study staff and researches who undertaking assessing outcomes were blinded on the treatment strategy.

### Outcomes

The primary outcomes were the absolute change of LDL-C and the rate of achieving targeted lipid control, specifically LDL-C <1.4 mmoL/L from baseline to 8 weeks. Other efficacy outcomes included total cholesterol (TC), triglycerides (TG), high-density lipoprotein cholesterol (HDL-C), apolipoproteins B (ApoB), apolipoproteins A1 (ApoA1), and lipoprotein(a) [Lp(a)] from baseline to 8 weeks. Exploratory endpoints reported herein included change in inflammatory biomarkers of high-sensitivity C-reactive protein (hs-CRP) and erythrocyte sedimentation rate (ESR) from baseline to 8 weeks.

Safety outcomes were the incidence of adverse events (AEs) and serious adverse events (SAEs) from baseline to 8 weeks. AEs encompass a range of occurrences, including but not limited to ALT or AST >3×ULN, general allergic reactions, localized injection site reactions, cognitive events, and musculoskeletal pain. SAEs included recurrent ischemic stroke (stroke or transient ischaemic attack), hemorrhagic stroke, myocardial infarction, cardiovascular death and all-cause mortality.

### Statistical analysis

Comparisons of baseline characteristics were performed using *t*-tests, Fisher exact tests, and chi-square tests. Continuous variables conforming to normal distribution were presented as mean ± standard deviation (SD), and in case of a skewed distribution as median and interquartile range (IQR). Categorical variables were described as counts and percentages. For safety outcomes, AEs and SAEs were summarized by treatment group using descriptive statistics with rate ratios. Analysis was based on the intention-to-treat (ITT) principle. At the same time, we performed interaction and stratified analyses from full-factorial mixed model based on age (<65 and ≥65 years); gender (male and female); statin treatment (yes and no) at baseline; and calculated LDL-C at baseline (<median and ≥median), which was in order to investigate the consistency of treatment effect across diverse patient subgroups by multivariate analysis of variance and *t*-tests methods. Tests are two-sided throughout and a *p*-value below 0.05 was considered as significant. Statistical analysis was performed using SPSS software version 20.0 (SPSS, Chicago, IL, United States).

## Results

### Cohort clinical characteristics

**Figure 1:**
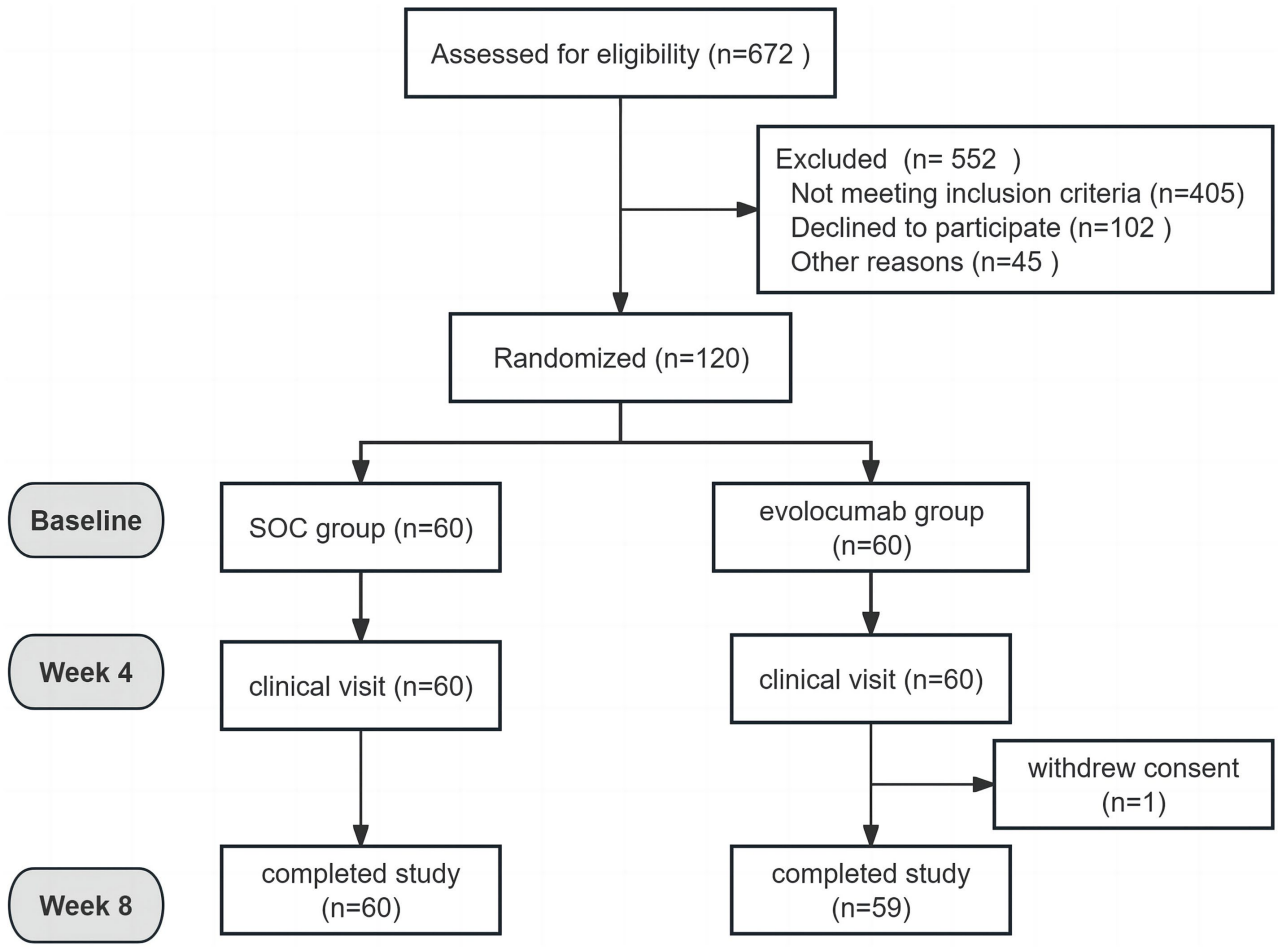
Study flowchart.

Totally, 120 eligible AIS patients were enrolled randomly 1:1 assigned to receive evolocumab combined with SOC treatment (*n* = 60) or sole SOC treatment (*n* = 60). The baseline characteristics of patients are shown in Table 1. The mean (SD) age of the participants in the evolocumab group was 60.97 (7.17) years and that in the SOC group was 62.88 (9.13) years. Two groups were well matched for age, gender, body mass index, history of diabetes mellitus, hypertension and CHD. Most patients were first onset [39 (65.0%) vs. 41 (68.33%)] and only a minority of patients [6 (10.0%) vs. 3 (5.0%)] were treated with statins before hospitalization within 4 weeks. While the evolocumab group had a higher proportion of current smoking compared with SOC group [25 (41.67%) vs. 13 (21.67%), *p* = 0.019]. Notably, patients in the evolocumab group had a significantly higher NIHSS score at baseline compared to the SOC group indicating higher stroke severity in evolocumab group (*p* = 0.022). The baseline LDL-C, TC, TG, HDL-C, ApoB, ApoA1, Lp(a), alanine aminotransferase (ALT) and aspartate aminotransferase (AST) levels did not differ significantly between two groups. One patient in the evolocumab group withdrew the trial for personal reason at the final visit at 8 weeks, occurring 119 patients participating in the final experiment (Figure 1). All the 120 patients received atorvastatin 40 mg/day and ezetimibe 10 mg/day at discharge, 116 patients (58 in the evolocumab group and 58 in the SOC group) received atorvastatin 40 mg/day and ezetimibe 10 mg/day at week 4 and week 8, without significant differences between groups.

**Table 1:** Baseline demographic and characteristics of the study patients.

	Evolocumab (*n* = 60)	SOC (*n* = 60)	*p*
Age, years	60.97 ± 7.17	62.88 ± 9.13	0.203^a^
Male gender, *n* (%)	46 (76.67)	38 (63.33)	0.111^b^
Body mass index, kg/m^2^	25.04 ± 2.99	24.03 ± 4.06	0.125^a^
Active smoking, *n* (%)	25 (41.67)	13 (21.67)	0.019^b^
Diabetes mellitus, *n* (%)	21 (35.0)	22 (36.67)	0.849^b^
Hypertension, *n* (%)	47 (78.33)	40 (66.67)	0.152^b^
History of CHD, *n* (%)	4 (6.67)	6 (10.0)	0.053^c^
First onset, *n* (%)	39 (65.0)	41 (68.33)	0.698^b^
Prior statin treatment, *n* (%)	6 (10.0)	3 (5.0)	0.268^c^
NIHSS score	3.28 ± 3.07	2.25 ± 1.51	0.022^a^
LDL-C, mmol/L	3.15 ± 0.96	3.17 ± 0.83	0.915^a^
TC, mmol/L	4.12 ± 1.30	4.22 ± 1.18	0.676^a^
TG, mmol/L	1.68 ± 1.07	1.61 ± 0.74	0.674^a^
HDL-C, mmol/L	1.04 ± 0.26	1.05 ± 0.30	0.895^a^
ApoA1, mmol/L	1.25 ± 0.25	1.25 ± 0.27	0.913^a^
ApoB, mmol/L	0.92 ± 0.35	0.98 ± 0.30	0.305^a^
Lp(a), mmol/L	88.40 ± 117.30	72.93 ± 96.86	0.432^a^
hs-CRP, mg/L	7.42 ± 12.07	15.06 ± 26.63	0.046^a^
ESR, mm/h	24.05 ± 16.79	24.33 ± 22.71	0.938^a^
ALT, U/L	19.77 ± 13.09	22.55 ± 16.40	0.306^a^
AST, U/L	19.17 ± 6.26	21.77 ± 9.19	0.073^a^

SOC, standard of care; CHD, coronary heart disease; NIHSS, National Institutes of Health Stroke Scale; LDL-C, low-density lipoprotein cholesterol; TC, total cholesterol; TG, triglycerides; HDL-C, high-density lipoprotein cholesterol; Apo, apolipoprotein; Lp(a), lipoprotein(a); hs-CRP, high-sensitivity C-reactive protein; ESR, erythrocyte sedimentation rate; ALT, alanine aminotransferase; AST, aspartate aminotransferase.

a *t-test.*

b Chi-square.

c Fisher exact test.

### Efficacy

**Figure 2:**
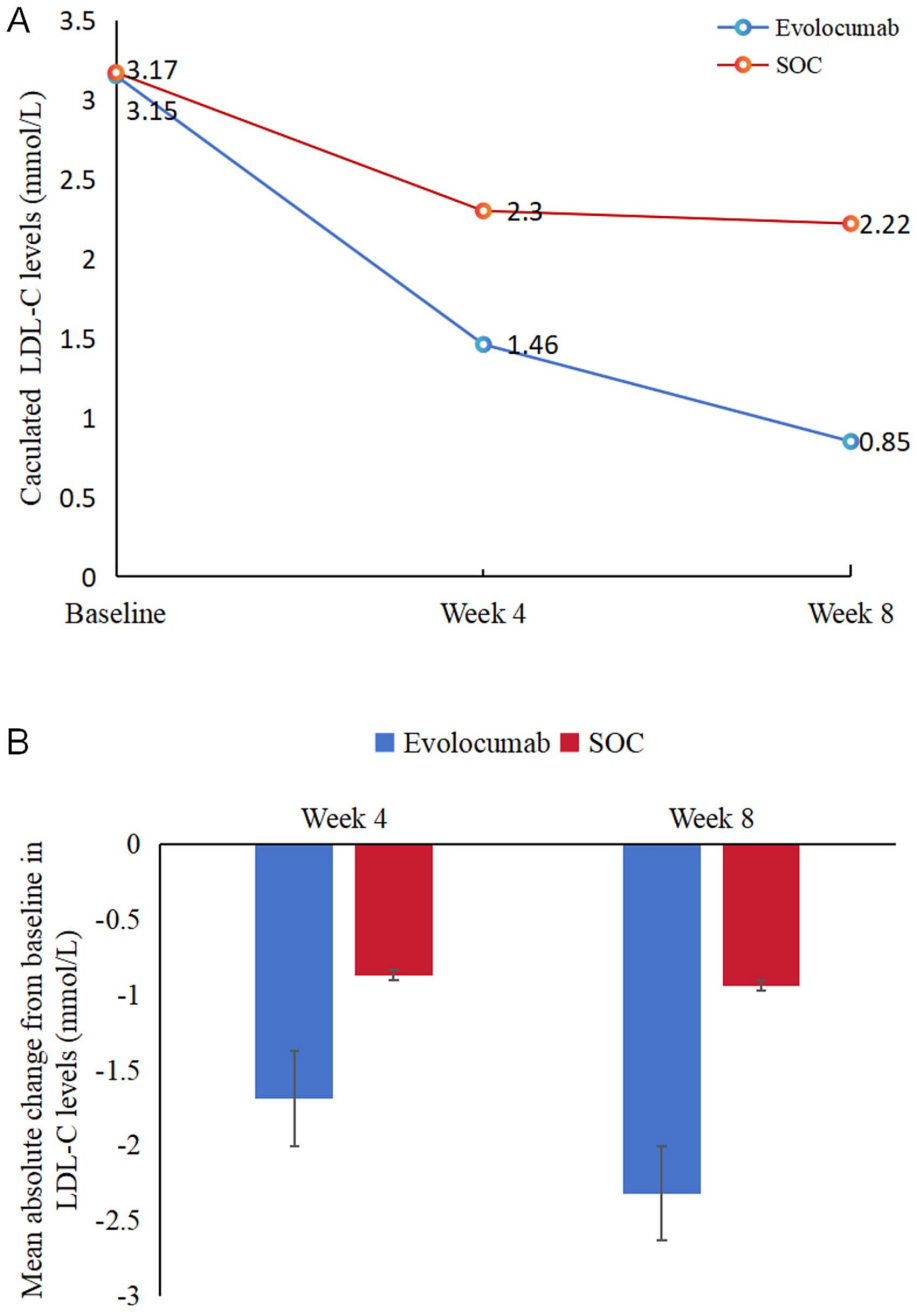
Changes in LDL-C levels over time. **(A)** Mean LDL-C levels at baseline, 4 weeks and 8 weeks in two groups. **(B)** Mean absolute changes in LDL-C from baseline to 4 weeks and 8 weeks in two study groups.

Calculated and change of LDL-C levels were available at baseline as well as at 4 and 8 weeks (Figure 2). Absolute change in calculated LDL-C from baseline to 8 weeks was −2.32 ± 0.92 in the evolocumab group (from a mean 3.15 mmol/L to 0.85 mmol/L) vs. −0.94 ± 0.90 in the SOC group (from a mean 3.17 mmol/L to 2.22 mmol/L), amounting to a mean difference of −1.37 between groups (95% CI = −1.70 to −1.04; *p* < 0.001) (Table 2). At 8 weeks, LDL-C was reduced to <1.4 mmol/L in 81.67% of patients in the evolocumab group as compared with 13.33% in the SOC group. However, the reduction in LDL-C levels was not evident at 4 weeks between two groups for all 120 patients with a mean difference of −0.83 (95% CI = −2.04 to 0.39) (*p* = 0.181). Interaction and stratified analyses of the primary endpoint revealed that LDL-C reductions were consistent in relation to age; gender; statin treatment history; and calculated LDL-C levels at baseline (all *p*-value for interaction >0.05; Table 3).

**Table 2:** Changes in lipid levels between baseline and during follow-up.

	Evolocumab (*n* = 60)	SOC (*n* = 60)	Mean difference (95% CI)	*p* ^a^
LDL-C (mmol/L)				
Baseline	3.15 ± 0.96	3.17 ± 0.83	−0.02 (−0.34 to 0.31)	0.915
Week 4	1.46 ± 4.56	2.30 ± 1.00	−0.84 (−2.04 to 0.35)	0.164
Week 8	0.85 ± 0.51	2.22 ± 0.77	−1.37 (−1.61 to −1.14)	<0.001
Absolute change from baseline to week 4	−1.69 ± 4.67	−0.87 ± 0.92	−0.83 (−2.04 to 0.39)	0.181
Absolute change from baseline to week 8	−2.32 ± 0.92	−0.94 ± 0.90	−1.37 (−1.70 to −1.04)	<0.001
LDL-C <1.4 mmol/L at week 8 (%)	49 (81.67%)	8 (13.33%)	—	<0.001
Other lipids absolute change from baseline to week 4 (mmol/L)				
TC	−1.81 ± 1.19	−0.39 ± 0.95	−1.41 (−1.80 to −1.02)	<0.001
TG	−0.28 ± 1.14	−0.18 ± 0.69	−0.11 (−0.45 to 0.23)	0.537
HDL-C	0.12 ± 0.25	0.06 ± 0.26	0.06 (−0.03 to 0.15)	0.193
ApoA1	0.07 ± 0.26	−0.05 ± 0.23	0.11 (0.02 to 0.20)	0.013
ApoB	−0.53 ± 0.31	−0.10 ± 0.21	−0.42 (−0.52 to −0.33)	<0.001
Lp(a)	−12.63 ± 33.28	1.68 ± 34.99	−14.31 (−26.65 to −1.96)	0.024
Other lipids absolute change from baseline to week 8 (mmol/L)				
TC	−1.84 ± 1.11	−0.40 ± 0.93	−1.44 (−1.82 to −1.07)	<0.001
TG	−0.31 ± 1.19	−0.10 ± 0.82	−0.21 (−0.58 to 0.16)	0.265
HDL-C	0.10 ± 0.19	0.10 ± 0.22	−0.001 (−0.07 to 0.07)	0.996
ApoA1	0.06 ± 0.25	0.001 ± 0.23	0.063 (−0.02 to 0.15)	0.154
ApoB	−0.52 ± 0.33	−0.13 ± 0.23	−0.39 (−0.49 to −0.28)	<0.001
Lp(a)	−24.53 ± 60.97	4.51 ± 40.96	−29.04 (−47.87 to −10.22)	0.003

SOC, standard of care; LDL-C, low-density lipoprotein cholesterol; TC, total cholesterol; TG, triglycerides; HDL-C, high-density lipoprotein cholesterol; Apo, apolipoprotein; Lp(a), lipoprotein(a).

a *t-test.*

**Table 3:** Subgroup analyses for the absolute change in LDL-C from baseline to 8 weeks.

	Evolocumab		SOC		Mean difference (95% CI)	*p* ^a^	Interaction *p*^b^
	*n* = 59	Mean ± SD	*n* = 60	Mean ± SD			
Age							0.148
<65 years	41	−2.28 ± 0.96	31	−0.65 ± 0.94	−1.64 (−2.09 to −1.19)	<0.001	
≥65 years	18	−2.42 ± 0.84	29	−1.27 ± 0.74	−1.14 (−1.61 to −0.67)	<0.001	
Gender							0.803
Male	45	−2.31 ± 0.96	38	−0.97 ± 0.96	−1.34 (−1.76 to −0.93)	<0.001	
Female	14	−2.36 ± 0.82	22	−0.92 ± 0.80	−1.44 (−2.00 to −0.87)	<0.001	
Statin history							0.842
Yes	6	−2.14 ± 0.68	3	−0.62 ± 1.25	−1.51 (−2.99 to −0.04)	0.046	
No	53	−2.34 ± 0.95	57	−0.97 ± 0.89	−1.38 (−1.72 to −1.03)	<0.001	
LDL-C at baseline							0.209
<The median	32	−1.73 ± 0.55	27	−0.42 ± 0.58	−1.31 (−1.61 to −1.02)	<0.001	
≥The median	27	−3.03 ± 0.76	33	−1.39 ± 0.88	−1.64 (−2.08 to −1.21)	<0.001	

SOC, standard of care; CI, confidence interval; LDL-C, low-density lipoprotein cholesterol.

a *t-test.*

b Multivariate analysis of variance.

Study also revealed that the levels of TC, ApoB and Lp(a) in evolocumab group gradually declined over time. However, it showed an upward trend for Lp(a) levels in SOC group. Trials observed greater absolute reductions of −1.84 ± 1.11 in TC (*p* < 0.001), −0.52 ± 0.33 in ApoB (*p* < 0.001) and −24.53 ± 60.97 in Lp(a) (*p* = 0.003) at week 8 in evolocumab group compared with SOC group. Compared to the placebo group, evolocumab significantly elevated ApoA1 levels at week 4 (*p* = 0.013), but these elevations were comparable between two groups at week 8 (*p* = 0.154) (Table 2).

### Safety

The percentage of patients experienced AEs and SAEs were similar between groups (Table 4). No case was considered SAEs resulting in study drug discontinuation. Liver function (ALT or AST increase >3× ULN) was the most common reported adverse event, occurring in two patients (3.33%) in the evolocumab and two patients (1.67%) in the SOC group (*p* = 0.56). Local injection site reaction was reported in two patients (3.33%) in evolocumab group and musculoskeletal pain was reported in one patient (1.67%) in SOC group. During the eight-week follow-up period, one patient in the SOC group experienced a recurrence of cerebral infarction with hemorrhage transformation.

**Table 4:** Adverse events.

	Evolocumab (*n* = 60)	SOC (*n* = 60)	*p* ^a^
Any adverse event	5	6	0.75
Serious adverse event	0	1 (3.33%)	0.32
Events of special interest			
ALT or AST >3×ULN	2 (3.33%)	2 (3.33%)	1.00
General allergic reaction	0	0	-
Local injection site reaction	2 (3.33%)	—	—
Neurocognitive event	1 (1.67%)	1 (1.67%)	1.00
Musculoskeletal pain	0	1 (1.67%)	0.32
Cerebrovascular event	0	1 (3.33%)	0.32
Cerebral hemorrhage	0	1 (3.33%)	0.32
Myocardial infarction	0	0	—
Cardiovascular death	0	0	—
All-cause death	0	0	—

SOC, standard of care; ALT, alanine aminotransferase; AST, aspartate aminotransferase.

a Fisher exact test.

### Inflammatory biomarkers

There was no significant difference of hs-CRP or ESR levels change between groups. Absolute change in hs-CRP from baseline to 8 weeks was −3.45 ± 12.41 in the evolocumab group vs. 0.64 ± 28.05 in the SOC group, amounting to a mean difference of −4.09 between groups (95% CI = −11.99 to 3.81; *p* = 0.307). Absolute change in ESR from baseline to 8 weeks was −1.15 ± 16.81 in the evolocumab group vs. −2.83 ± 22.56 in the SOC group, amounting to a mean difference of 1.68 between groups (95% CI = −5.55 to 8.91; *p* = 0.646).

## Discussion

In recent years, the optimizing lipid management of AIS has been a focus of intensive research and clinical interest, given its significant impact on morbidity and mortality worldwide (25). To our knowledge, this study was the first reported trial exploring the effectiveness and safety of evolocumab on a high-intensity statin and ezetimibe therapy background in patients presenting with AIS initiated in-hospital. This study showed that treatment with evolocumab 420 mg every 4 weeks with atorvastatin 40 mg/day and ezetimibe 10 mg/day significantly reduce the LDL-C levels compared with atorvastatin 40 mg/day and ezetimibe 10 mg/day alone among the AIS patients. Evolocumab lowered mean LDL-C levels from 3.15 mmol/L to 1.46 mmol/L as early as 4 weeks, and enabled >80% of patients to achieve guideline-recommended LDL-C targets at 8 weeks. During the brief duration of the study, the treatment was well-tolerated, with no notable imbalances in AEs, which aligned with the safety and tolerability profiles reported in previous studies involving evolocumab in more controlled clinical environments.

Evolocumab reduces the degradation of low-density lipoprotein receptor by inhibiting the function of PCSK9, so that more LDL-C can be taken up and cleared by the liver, thereby reducing plasma LDL-C levels (26). This mechanism may help slow or stop the progression of atherosclerotic plaque and reduce the risk of plaque rupture (27). In addition, many studies had also shown that PCSK9 was associated with neuronal apoptosis and synaptic plasticity (28, 29). Therefore, we hypothesized evolocumab may also have a role in protecting neurons and promoting nerve regeneration and repair, thereby promoting the recovery of nerve function after AIS. Some preliminary clinical observations suggested that early use of evolocumab after AIS, in combination with standard treatments such as antiplatelet agents and thrombolytic therapy, may improve neurological outcomes. Early initiation of PCSK9 inhibitors reduced LDL-C 30% more than ezetimibe, and 60% more than placebo when added to statins, accompanied by a decrease in the risk of major cardiovascular and cerebrovascular events (30). Furthermore, real-world data in very high-risk arteriosclerotic cardiovascular disease patients with AIS in China suggested that the proportion of reaching the target of lowering LDL-C levels was 44.91% in the evolocumab (140 mg every 2 weeks) on a background of statin compared with the 3.12% of SOC-treated patients, as well as the former with the lower incidence of recurrent cerebrovascular events (31). A retrospective study indicated that PCSK9 inhibitors in AIS patients undergoing post-mechanical thrombectomy may lead to improved discharge outcomes and decrease the occurrence of hemorrhage and early neurologic deterioration (32). Meta-analysis of recent randomized clinical trials revealed promising results regarding the efficacy of PCSK9 inhibitors reducing the incidence of stroke by 25%, highlighting the potential of PCSK9 inhibitors as valuable additions to the current armamentarium for cerebrovascular risk reduction (33, 34).

PCSK9 inhibitors have also been reported to be associated with the regulation of Lp(a). Lp(a), a lipoprotein that causes atherosclerosis, has been determined as an independent risk factor of arteriosclerotic cardiovascular disease in addition to LDL-C (35). Currently, there are relatively few treatment options for Lp(a) (36). A meta-analysis showed that PCSK9 monoclonal antibodies significantly reached a reduction of 21.9% of circulating Lp(a) levels, and that the higher the baseline Lp(a) level, the greater the reduction and the greater the benefit, providing more options for reducing Lp(a) (37). A study has indicated that increased serum Lp(a) levels served as a predictor for the likelihood of early stroke recurrence among patients who have experienced their first-ever ischemic stroke (38). Certain individuals with elevated Lp(a) levels might reap the benefits of a more rigorous statin treatment aimed at reducing LDL-C concentrations, albeit studies have demonstrated that statin therapy may also mildly elevate Lp(a) levels (39). Consistent with prior research, our investigation revealed that Lp(a) levels in AIS patients escalated in tandem with an extended duration of statin and ezetimibe administration. Conversely, Lp(a) concentrations diminished among patients receiving evolocumab. This finding offered evidence for therapeutic alternatives aimed at reducing Lp(a) levels, which was anticipated to further mitigate the risk of cardiovascular and cerebrovascular disease.

Recent studies have suggested that PCSK9 inhibitors may have potential immunomodulatory properties, including modulating inflammatory signaling pathways and reducing inflammatory cell infiltration (40). During the initial stages of an ischemic stroke, the release of danger-/damage-associated molecular patterns by injured and dying neurons initiates microglial activation, which subsequently triggers a peripheral immune cell response (41). Patients treated with PCSK9 inhibitors exhibited a reduced expression of pro-inflammatory proteins within the plaque (21). However, evolocumab in our clinical trial did not show to reduce hs-CRP and ESR. Whether evolocumab exhibits an anti-inflammatory mechanism and its precise mode of action in patients with AIS remains a subject of ongoing investigation.

This study had several limitations. First, it was a single-center study design with a limited sample size, short study duration and no blindness, which might cause bias to results. Our study included only individuals living in a city in China, limiting our results’ applicability to the whole of Chinese patients. Second, although the baseline lipids in our study matched in two groups, many factors such as vascular stenosis and antiplatelet drug cannot be fully controlled. Third, in comparison to prior clinical studies conducted in other countries (4), the LDL-C level observed at baseline in our study was significantly higher. Since only 7.5% of them were treated by lipid-lowering drugs before, which might be owing to individuals in our living in a less prosperous city in China and patients lacked adequate awareness about lipid management. Compared to statins or ezetimibe, evolocumab is more expensive and not recommended as a first-line treatment. While the high cost can limit accessibility, we believe that understanding its potential benefits could inform future healthcare strategies. Nevertheless, how to accurately identify the patient population suitable for use with evolocumab is a key issue.

## Conclusion

Based on our available preliminary data, early use of evolocumab in patients with AIS had shown significant LDL-C reduction and was well tolerated, which indicating broad clinical application prospect in the treatment for AIS. For this study is still going on, in the future, we are expected to have a better understanding of the status and role of evolocumab in the treatment of AIS.

## Data Availability

The raw data supporting the conclusions of this article will be made available by the authors, without undue reservation.
